# Sensitivity to the temporal structure of rapid sound sequences — An MEG study

**DOI:** 10.1016/j.neuroimage.2015.01.052

**Published:** 2015-04-15

**Authors:** Lefkothea-Vasiliki Andreou, Timothy D. Griffiths, Maria Chait

**Affiliations:** a UCL Ear Institute, 332 Gray's Inn Road, London WC1X 8EE, UK; b Wellcome Trust Centre for Neuroimaging, University College London, London WC1N 3BG, UK; c Institute of Neuroscience, Newcastle University Medical School, Newcastle upon Tyne NE2 4HH, UK

**Keywords:** Time perception, Magnetoencephalography, MMN, Offset response, Omission response, Auditory scene analysis, entrainment

## Abstract

To probe sensitivity to the time structure of ongoing sound sequences, we measured MEG responses, in human listeners, to the *offset* of long tone-pip sequences containing various forms of temporal regularity. If listeners learn sequence temporal properties and form expectancies about the arrival time of an upcoming tone, sequence offset should be detectable as soon as an expected tone fails to arrive. Therefore, latencies of offset responses are indicative of the extent to which the temporal pattern has been acquired. In Exp1, sequences were isochronous with tone inter-onset-interval (IOI) set to 75, 125 or 225 ms. Exp2 comprised of non-isochronous, temporally regular sequences, comprised of the IOIs above. Exp3 used the same sequences as Exp2 but listeners were required to monitor them for occasional frequency deviants. Analysis of the latency of offset responses revealed that the temporal structure of (even rather simple) regular sequences is not learnt precisely when the sequences are ignored. Pattern coding, supported by a network of temporal, parietal and frontal sources, improved considerably when the signals were made behaviourally pertinent. Thus, contrary to what might be expected in the context of an ‘early warning system’ framework, learning of temporal structure is not automatic, but affected by the signal's behavioural relevance.

## Highlights

• MEG brain responses to offsets (cessation) of long sound sequences are measured.
• Sound sequences are rapid, characterized by various forms of temporal patterning.
• Latencies of sequence offset responses are measures of sensitivity to temporal patterns.
• Temporal coding improves when signals are behaviourally pertinent.
• Temporal coding is supported by temporal, parietal and frontal sources.

## Introduction

Sensitivity to the timing of sensory input plays a crucial role in the perception of, and efficient interaction with, the environment (Nobre et al., 2007). Due to the dynamic nature of sound, temporal information is perhaps especially relevant in the context of the auditory modality. Music or speech are often a prime example, but much more generally, the ability to make sense of acoustic scenes, including resolving the identity and current state of objects, requires the capacity to extract and retain the temporal patterning of the stimulus sequence.

Tapping studies (see Repp, 2005 for review), a classic method for investigating sensitivity to temporal structure in sound, demonstrate that listeners can entrain to sequences of tones over a wide range of tempi and rhythmic patterns. The tapping profile often exhibits predictive properties — taps do not constitute reactions to a heard tone, but actually precedes the pacing — suggesting that listeners have internalized the pattern. In isochronous sequences, synchronization is commonly observed for tone inter-onset-intervals in the range 150–1800 ms (Fraisse, 1982; Repp, 2005) but the lower limit might be set by motor, rather than perceptual, constraints.

To understand how sensitivity to temporal structure might aid listening, investigations have focused on the effect of temporal context on processing, demonstrating that context-induced temporal orienting — when the stimulus history reliably predicts the timing of the next event — speeds up reaction time and improves discrimination/identification performance even in the absence of explicit attention (Jaramillo and Zador, 2011; Ellis and Jones, 2010; Lange, 2013; Tavano et al, 2014). This evidence is taken to support the hypothesis that the brain entrains to rhythmic sequences and recent imaging and electrophysiology studies have demonstrated effects consistent with this notion, implicating a network of temporal, parietal and motor regions in this process (Grahn and Rowe, 2009; Lakatos et al., 2013; Fujioka et al., 2012; Arnal and Giraud, 2012).

An ‘extreme’ hypothesis, motivated by a supposed ‘early warning system’ role for the auditory modality (Bendixen et al., 2012) suggests that any regular temporal structure should, in principle, be learnable. However, most previous investigations have focused on relatively slow rhythms (< 3 Hz) and simple (isochronous) temporal sequences (Lange, 2013; Schwartze et al., 2011; Fujioka et al., 2012). The degree to which the brain can ‘acquire’ more complex or rapid temporal patterns remains poorly understood. Outlining the operational limits of these mechanisms – that is, identifying the temporal patterns the brain is vs. is not sensitive to – is essential to uncovering the neural computations underlying sensitivity to time.

To systematically probe these mechanisms, we use an implicit timing paradigm, based on measuring brain responses to the *offset* (termination) of long sound sequences containing various temporal regularities. If the brain is able to learn the temporal structure of a sequence and form expectancies about the arrival time of an upcoming tone, sequence offset should be detectable as soon as an expected tone fails to arrive. The analysis of latencies of sequence-offset responses may thus provide a measure of the fidelity with which sequence time structure has been acquired and, consequently, a window onto the brain processes involved in tracking auditory input across time.

The ‘offset’ paradigm is notionally analogous to the ‘omission MMN’ paradigm which involves measuring brain responses to occasionally omitted events within on-going sequences (Yabe et al., 1997; Horváth et al., 2010; Bendixen et al., 2012; Jongsma et al., 2004; Hughes et al., 2001). However much of this work has focused on slow isochronous sequences and, in most cases, did not specifically measure latency (but see Karamürsel and Bullock, 2000; Yamashiro et al., 2009; Motz et al., 2013).

Emerging from this literature is the finding that brain responses to omitted sounds are initially (~ 50 ms) similar to presented tones, with a mismatch response emerging thereafter (Janata, 2001; Bendixen et al., 2009; SanMiguel et al., 2013), namely, that a template of a response to a predicted tone is ‘pre-activated’ at the time of its expected onset. In an intracranial recording study, Hughes et al. (2001) identified neural foci showing responses to both present and omitted tones as well as foci that appeared selective for omitted tones only (i.e. showed little response to actually presented tones) but found no instances of veridical responses (responses only to actually presented tones), suggesting that the expectation of future events is closely linked to processing of those actually presented.

The present series of MEG experiments explicitly focuses on the process of temporal expectation by measuring brain responses to non-arriving sounds within varying, increasingly complex temporal contexts. Importantly, and in contrast to much of the existing work in the field, the acoustic sequences used here are characterized by rapid rates (> 4 Hz) in order to specifically tap the brain mechanisms and computations sub-serving sensitivity to timing within the range relevant to sounds commonly encountered in our surroundings (e.g. the phonemic and syllabic rate of speech, animal vocalizations, etc). Focusing on brain responses evoked by the offset of a sequence, rather than occasional tone omissions, enables the isolation of activations specifically related to the non-arriving tone, devoid of overlap with responses to subsequent events (this is especially problematic for rapid sequences). Arguably, this approach is also more ecologically relevant — abrupt offsets in the environment are a potential signal for imminent danger and likely to engage rapid and efficient processing by the auditory system.

We hypothesized that the temporal structure of rapid isochronous tone-pip sequences is ‘automatically’ acquired by the brain, even in the absence of explicit attention. This is confirmed in Experiment 1 which demonstrates robust offset responses with a latency that is precisely related to when the non-arriving tone was expected to occur. Experiment 2 then tested simple, regularly repeating, but non-isochronous sequences and revealed significantly increased offset response latencies — indicating markedly worsened sensitivity to temporal structure in these stimuli. Experiment 3 tested, and confirmed, the hypothesis that offset response latencies would shorten when the sequences are attended, demonstrating that the implicit learning of temporal structure is not automatic but affected by behavioural relevance.

## Materials and methods

### Subjects

Ten subjects (mean age 27.6 years, SD = 4; 4 females) participated in *Experiment 1*. Fifteen subjects (mean age 26.6 years, SD = 5; 7 females) participated in *Experiment 2a* and an additional group of 5 subjects (mean age 25.6, SD = 6.1; 3 females) participated in *Experiment 2b*. Twelve subjects (mean age 25.6 years, SD = 4.9; 5 females) participated in *Experiment 3*. Experiment 1 shared five participants with Experiment 2a, one with Experiment 2b and two with Experiment 3. Experiment 2a shared five participants with Experiment 2b and seven with Experiment 3. Experiment 2b had five participants in common with Experiment 3. The experiments took place several weeks apart. All subjects were right-handed (Oldfield, 1971), reported normal hearing, normal or corrected to normal vision, and had no history of neurological disorders. The experimental procedures were approved by the research ethics committee of University College London and written informed consent was obtained from each subject. Subjects were paid for their participation.

### Stimuli

**Fig. 1:**
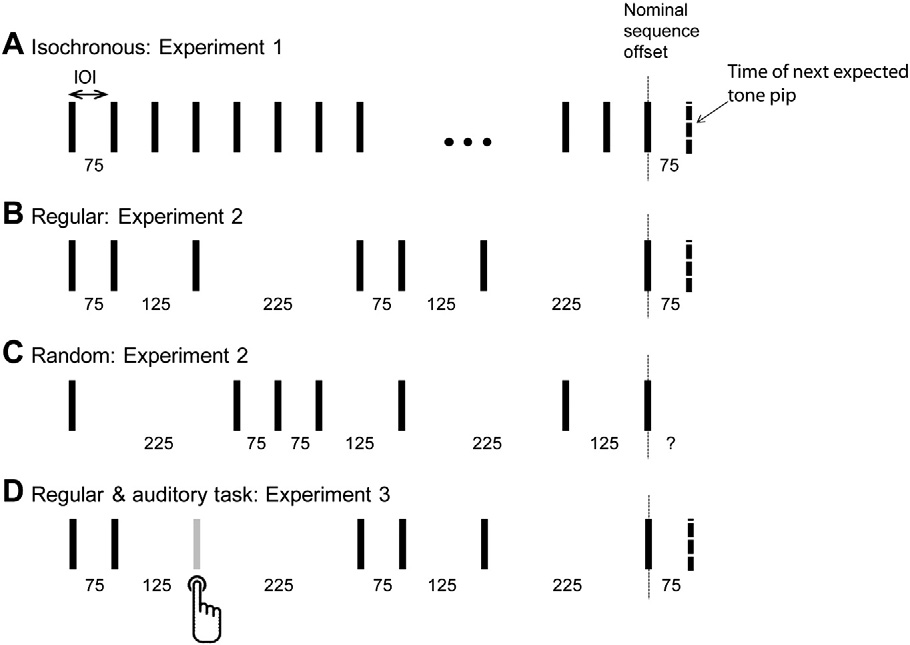
Schematic representation of the stimuli. Experiment 1 (A) consisted of isochronous sequences with one of 3 IOI durations (75, 125 or 225 ms). Experiment 2 contained two types of stimuli: Regular sequences, consisting of a sequential repetition of the 3 IOIs (B), and random sequences (C) where the IOIs were presented in random order. Experiment 3 consisted of the same regular sequences as in Experiment 2 except that these contained occasional frequency deviants which listeners were instructed to detect (D). The sequences were interrupted after a variable duration and brain responses were grouped for analysis according to the IOI expected after sequence interruption. The sequences plotted, save for the random sequence in C, are conditions where the expected IOI, if listeners are able to learn the temporal structure, is 75 ms. The dashed line marks the time of the next expected tone pip.

Fig. 1A schematizes the stimuli used in *Experiment 1*. The signals were sequences of 25 ms tone bursts (500 Hz) separated by silent intervals of a fixed duration, resulting in an isochronous rhythm. In different conditions, the duration of the silent interval was set to one of three values (50, 100 or 200 ms), corresponding to inter-onset-intervals (IOI) of 75, 125 and 225 ms, respectively. These IOI durations were chosen because rapid temporal patterns remain under-investigated, despite the fact that the temporal properties of many natural sound sequences are within this range. The sequences were of variable overall duration, consisting of a minimum of 24 and maximum of 36 tone-bursts. The stimulus set also contained long pure-tone stimuli (‘CONT’), used to measure the latency of ‘simple’ auditory cortical offset responses. The length of the constant tone varied randomly between 1500 and 3500 ms. The signals were created off-line at a sampling rate of 44.1 kHz and saved in a 16-bit stereo WAV format. In total, 150 repetitions of each of the 4 experimental conditions were presented. All conditions were presented in random order with the inter-sequence interval (ISI) randomized between 750 and 1500 ms.

*Experiment 2a* consisted of two stimulus blocks, order counter-balanced across participants. The first block (Fig. 1B) comprised of non-isochronous, but temporally regular, sequences. These were created by using the same IOIs as in the isochronous sequences (Experiment 1) but such that they alternated regularly between the 3 values e.g. 75–125–225–75–125–225 etc or 225–125–75–225–125–75 and so on. IOIs were presented with equal probability. The resulting sequences were therefore not strictly metrical (metrical rhythm, common in western music, is characterized by time values in the sequences constituting multiples of a fixed beat; Povel and Essens, 1985; Povel and Okkerman, 1981). As in Experiment 1, sequences consisted of a random number of tone bursts and were grouped into conditions based on the duration of the silent interval after the last tone, i.e. based on the time at which the next (non-arriving) tone is expected (see Fig. 1B). The ‘CONT’ condition, identical to that in Experiment 1, was also included in the stimulus set. The second block (Fig. 1C) comprised of temporally irregular sequences. The stimulus was generated as described for the regular sequences except that the order of the IOIs was randomly permuted, resulting in sequences that contained equal proportions of each of the three IOI values, but in random order. Fig. 1C schematizes an example of such a random sequence. The ‘CONT’ condition, identical to that in Experiment 1, was also included in the stimulus set. To facilitate learning of the patterns/statistics, each block began with a 2 minute long, uninterrupted presentation of the regular or random sequence. To assess potential effects of sequence complexity, the experiment was repeated (*Experiment 2b*) on a control group of 5 subjects using a single permutation of the three IOIs (i.e. 75–125–225–75–125–225 and so on).

Fig. 1D schematizes the stimuli used in *Experiment 3*. The stimuli were identical to those in the regular block of Experiment 2 (IOI order: 75–125–225–75–125–225 etc.) except that standard tone-pips were occasionally replaced by frequency deviants (‘targets’) which subjects were required to detect as quickly as possible. The frequency difference between the target and standard tones was set just above threshold (determined individually for each listener, mean frequency of deviant tones was 540.2 Hz, SD = 10.5). 25% of sequences in the stimulus set contained a target which could occur anywhere in the sequence. Trials that contained targets within 2 s of sequence offset were excluded from the analysis. Listeners received feedback about their performance at the end of each block (hits, misses and false positives). As in Experiment 2, above, the block began with a 2 minute long uninterrupted presentation of the regular sequence. This sequence contained no deviants.

In all three experiments, subjects were also presented with visual stimuli which consisted of landscape images, grouped in series of 3 (duration of each image was 5 s, with a 2 s between-series interval during which the screen was blank). Subjects were instructed to fixate at a cross, drawn in the middle of the display and press a button whenever the third image in a series constituted a repetition of the first or second one. Such repetitions occurred in 10% of the trials. The visual task served as a decoy task — a means to ensure subject's alertness and (in Experiments 1 and 2) to divert attention away from the auditory stimuli. At the end of each block subjects received feedback about their performance (number of hits, misses and false positives). Performance was at ceiling — Experiment 1:94.2% (SD = 7.2), Experiment 2: 96.5% (collapsed over Exp2a and Exp2b; SD = 5.7), Experiment 3: 90.2% (SD = 9.6).

### Procedure

The MEG scans were conducted in a seated upright position in a magnetically shielded room. The signals were delivered binaurally with a tube phone attached to ear plugs (E-A-RTONE 3A 10 Ω, Etymotic Research, Inc) inserted into the ear canal and presented at a comfortable listening level adjusted individually for each participant. The experiment proper was preceded by a functional source-localizer recording in which subjects listened to 200 repetitions of a 1-kHz, 50-ms sinusoidal tone (ISI randomized between 750 and 1550 ms). These responses were used to verify that the subject was positioned properly in the machine, that signals from auditory cortex showed a satisfactory signal-to-noise ratio (SNR), and to determine which MEG channels best reflected activity within auditory cortex. The experiment proper lasted for about 1 h and involved naive listeners passively listening to sounds while performing an irrelevant (decoy) visual task. Responses were executed using a button box held in the right hand. Short breaks were provided every 10 min but subjects were required to remain still. Their position was monitored at the beginning and end of each run.

In Experiment 3, in addition to the visual task, subjects also performed an auditory task, as described above. Responses were delivered using a different button on the same button box and feedback was provided at the end of each block.

### Neuromagnetic recording and data analysis

Magnetic signals were recorded using a CTF-275 MEG system (axial gradiometers, 274 channels, 30 reference channels; VSM MedTech, Canada). Data were acquired continuously with a sampling rate of 300 Hz and a 100 Hz hardware low pass filter.

Functional localizer data were divided into 700-ms epochs, including 200-ms pre-onset, and baseline-corrected to the pre-onset interval. The M100 onset response (Roberts et al., 2000) was identified for each subject as a source/sink pair in the magnetic-field contour plots distributed over the temporal region of each hemisphere. The M100 current source is generally robustly localized to the upper banks of the superior temporal gyrus in both hemispheres (Lütkenhöner and Steinsträter, 1998). For each subject, the 40 strongest channels at the peak of the M100 (20 in each hemisphere) were considered to best reflect activity in the auditory cortex, and these were selected for the RMS (root mean square) analysis below.

In the main experiment, the evoked response analysis was time locked to the offset of the last tone of a sequence. The analysis epochs were 1750 ms duration, including a 1000 ms pre-offset period. The data were baseline corrected to the 600–750 ms post-offset period (no sounds were present in that interval) and low-pass filtered at 30 Hz. A denoising source separation (DSS) procedure was employed, over data from all 274 sensors, to find the most reproducible linear combination of sensors across trials (de Cheveigné, 2010; de Cheveigné and Parra, 2014). The first DSS component, i.e. the most reproducible component in the recorded brain activity, was projected back to the data and used for subsequent analysis. Since the analysis is based on a single DSS component, the temporal dynamics of the response are identical across channels (channels only differ in overall amplitude). In order to meaningfully summarize the data across subjects and for display purposes, we chose to present the data from the auditory channels as these generally exhibited the highest amplitude. Source analysis (see below) was based on all channels.

The individual RMS (root mean square) time series of the field strength across the 20 best auditory channels for each hemisphere (determined by the functional localizer), was calculated for each condition and participant. The time course of the RMS, reflecting the instantaneous amplitude of neural responses, is employed as a measure of the dynamics of the brain response. The congruity of the time course of activation across subjects was evaluated using the bootstrap method (Efron and Tibshirani, 1993; 1000 iterations, balanced), based on the individual RMS time series.

For illustration purposes, the group-RMS (RMS of individual subject RMSs) time series are plotted, but statistical analysis was always performed on peak latencies extracted from each subject's data. Latencies of offset responses were estimated by determining, for each participant and condition, the latency corresponding to the maximum value within a 100 ms window around the grand-RMS peak. As a measure of cross-subject variability, latency histograms were computed by an iterative bootstrap-based procedure where, on each iteration, RMS time series of 5 subjects were randomly chosen (with replacement), the grand-RMS computed, and the latency of the relevant peak determined based on a 100 ms window defined around the grand-RMS peak of the full data set. The iterative process (1000 iterations) generated latency histograms (Fig. 4B, bottom) from which the mean latency and its variability can be estimated. Occasionally, when a peak is not present (e.g. this occurred for some subjects in Experiment 2) the peak latency determination procedure would select a point at the edge of the window. This was allowed, and is reflected in higher variability, across subjects (larger error bars or wider distributions), in the relevant conditions (e.g. IOI225 condition in Experiment 2).

Offset response latencies in Experiment 1 (see Results) are used as a benchmark against which data from Experiments 2 and 3 are compared. Statistical analysis involves mixed design repeated measure ANOVAs with IOI condition as a within-subject measure and experiment as between-subject measure. These are followed by (Bonferroni corrected) post hoc analyses to assess any significant main effects. The Greenhouse–Geiser correction was used when required. Homogeneity of variance was assessed with Levene's test. The single instance in which this test indicated potential differences is between Exp2a and Exp2b (as expected due to the very different subject numbers; and as evident from Fig. 3). In all other cases there was no evidence of differences (p > 0.133 for all).

**Fig. 2:**
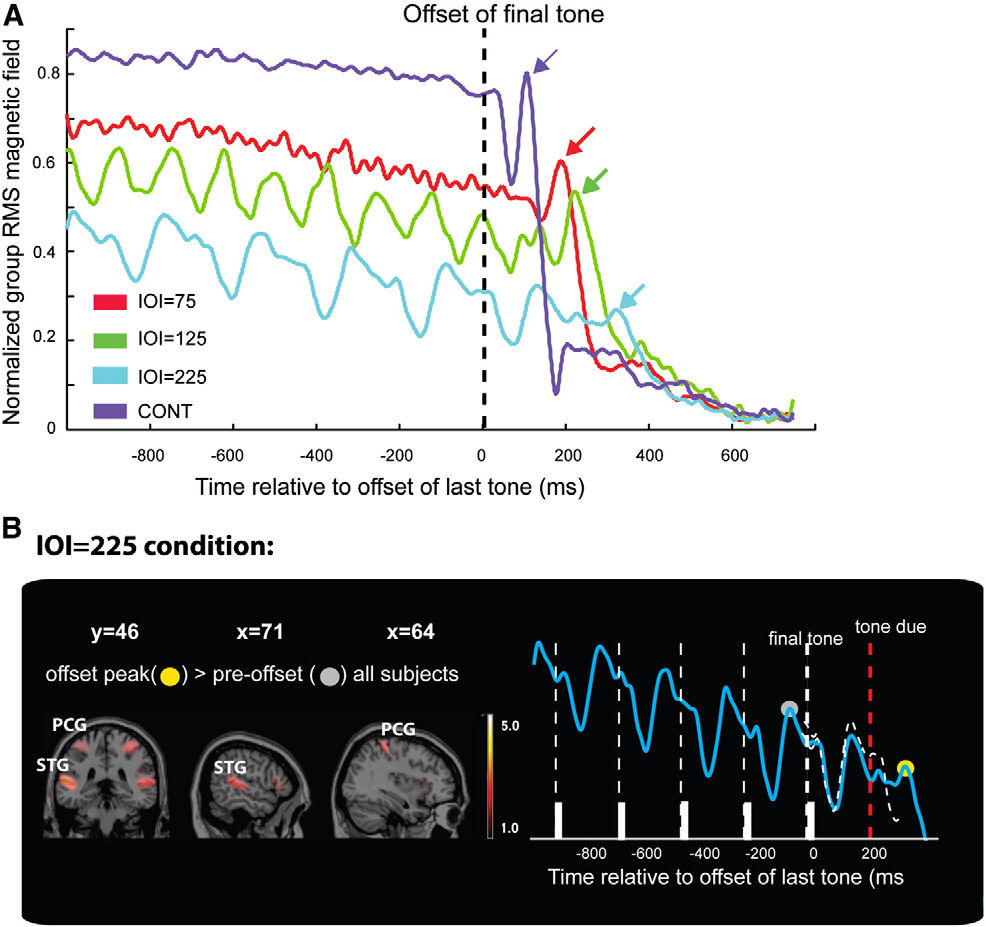
Results of Experiment 1. A: Measured brain responses (group RMS), time-locked to the offset of the last tone (0 ms). Offset responses are indicated with arrows. B: Focus on the IOI = 225 ms condition. Right: evoked response. White bars indicate the ultimate 5 tones in the sequence. An offset response (yellow circle) is generated shortly after the expected time of arrival of the missing tone (red dashed line). The response to the last audible tone is reproduced over the response to the missing tone (white dashed curve) to facilitate comparison of response dynamics. Source localization results for that condition are on the left. Plotted are t-maps overlaid on a ch2.nii.gz atlas. Significant clusters for the offset peak > pre-offset (indicated by grey dot) are in superior temporal gyrus (STG) and post-central gyrus (PCG), bilaterally. See also Table 1.

To ‘blindly’ identify the sources of the evoked activity, the multiple sparse priors (MSP) method, with group constraints, was used (Friston et al., 2008; Litvak and Friston, 2008; Litvak et al., 2011). Analysis was based on raw time-averaged data from all channels, low-pass filtered at 48 Hz. The inversion time window encompassed the entire epoch. Subsequently, two 50 ms time intervals were defined for analysis: (i) around the grand-average offset peak (see yellow circle in Fig. 2) and (ii) around a pre-offset interval (100 ms before sequence offset; see grey circle in Fig. 2). This enabled the identification of sources active during sequence processing, during offset processing, and those activated more during offset than while listening to the ongoing pattern (or vice versa). The resulting source estimates were averaged over that interval, projected to a three-dimensional source space, smoothed (isotropic Gaussian kernel of 5 mm full-width at half-maximum) to create images of source activity for each subject, and then taken to the 2nd level. The statistical analysis was conducted using the general linear model as described by Friston et al. (1995). The results were overlaid on a ch2.nii.gz atlas using MRIcron software (http://www.mricro.com/mricron/install.html). The brain areas that correspond to the stereotactic Montreal Neurological Institute (MNI) coordinates were identified using xjView toolbox (http://www.alivelearn.net/xjview8/).

## Results

The analysis is focused on the *latency* of offset responses (‘when does the brain ‘realize’ that the sequence has ended?’), a measure relatively neglected in previous work. As discussed above, if the brain is able to learn the structure of the ongoing sequence an offset (non-arrival of an expected event) will be detected more rapidly (and with greater consistency across trials). Hence we argue that offset response latencies should be a major parameter of interest when probing sensitivity to time. Comparing latency across conditions enabled us to test straightforward predictions about whether and how the auditory brain represents sequence timing and also affords a clear measure of computational efficiency. For descriptive purposes group RMS data (across subjects) are presented (Figs. 2, 4A) but the statistical analysis is performed on peak latencies measured from each subject's data as described in the Materials and methods section.

### Experiment 1 — unattended isochronous sequences

The stimuli in Experiment 1 were isochronous tone-pip sequences with inter-onset intervals (IOI) of 75, 125 or 225 ms. The stimulus set also contained long pure tone stimuli (‘CONT’), used to estimate the latency of simple offset responses. Fig. 2A shows the recorded brain responses to each of the 4 experimental conditions, time-locked to stimulus offset (dashed line; this corresponds to the offset of the long pure tone in the CONT condition or the offset of the last tone-pip in the tone-pip sequences). Plotted is a 1750 ms epoch from 1000 ms before to 750 ms after the offset. Responses to all stimuli show a gradual decrease in amplitude, attributable to the effects of adaptation, suggesting that these processes continuously shape brain responses even in relatively long sound sequences. The response to the offset of a long pure tone (CONT) is manifested as an abrupt drop in the sustained response which is followed by an ‘offset peak’ (see purple arrow). Responses to the offset of the tone sequences show similar, but delayed, patterns (see red, green, blue arrows). In the slower sequences (IOI125 and IOI225) responses to individual tones are visible in the pre-offset interval, with a prominent ‘offset peak’ arising around the time the non-arriving tone is expected to occur. These responses demonstrate that the auditory brain codes the offset of sound patterns (‘second order transient’) in addition to onsets and offsets of single tones (see also Yamashiro et al., 2009; Hughes et al., 2001).

This work focuses on the latencies of these offset responses. An ideal observer is expected to detect the offset of the sequence once the appropriate silent duration has elapsed. The measured responses reveal such a pattern with offset peaks occurring progressively later in time. The mean latencies are: CONT = 95 ms, IOI75 = 195 ms, IOI125 = 227 ms, IOI225 = 317 ms. To evaluate IOI learning, the relevant silent duration was subtracted from the measured offset-peak latency, resulting in a “*corrected*” offset latency for each condition (see Fig. 4B; orange bars). These latency data will be used as a benchmark against which to compare data from the non-isochronous sequences, in Experiments 2 and 3.

An ideal observer account would predict that, after the correction, all offset response latencies should be identical. This pattern is partially observed: A repeated measures ANOVA showed a main effect of condition (F(1.457,13.115) = 13.401; p < 0.001). Post hoc, Bonferroni corrected, pairwise comparisons indicated a significant difference between CONT and IOI75 (p < 0.0001), CONT and IOI125 (p = 0.006) and IOI75 and IOI125 (p = 0.001). There was no significant difference between IOI225 and the other conditions (p = 0.194, 0.125 and 1.00 respectively). While the statistics are not entirely conclusive on this point, there appears to be an overall ~ 25 ms delay between the corrected latencies of the different IOI conditions and that of CONT (see dashed line in Fig. 4B). This potentially reflects the extra computational demands required for detecting the offset of a sequence (reacting to an expected, but non-arriving tone-pip) relative to the much simpler task of detecting the drop in power associated with the termination of a continuous pure tone.

Fig. 2B focuses on the slowest condition (IOI225) as this is closest to the time scales studied in recent investigations (Fujioka et al., 2012; Lakatos et al., 2013). The timing of the 5 last tones in the sequence are marked on the x-axis and the brain data demonstrate reproducible response dynamics to each tone, commensurate to those reported for longer IOIs (390 ms in Fujioka et al., 2012; 600 ms in Lakatos et al., 2013). When a tone fails to arrive (red dashed line), the drop in amplitude, which always occurs shortly after tone onset does not ensue and an offset peak is generated soon after (yellow dot). A response to the last tone in the sequence (dashed grey line) is reproduced above the ‘missing tone’ response to facilitate comparison of these temporal dynamics.

MSP source analysis was used to ‘blindly’ identify the neural substrates involved in coding the tone sequence. The analysis was applied at two time points — offset (location indicated by the yellow dot in Fig. 2B) and at pre-offset (grey dot in Fig. 2B), before the arrival of the last tone. The group data are shown in Fig. 2B (see also Table 1 for coordinates and t values). The analysis identified 2 cortical foci as contributing to the offset peak: The Superior temporal gyrus (STG) in auditory cortex, and the post central gyrus (PCG) in the parietal lobe. Activation in those areas contributed to coding the on-going sequence (pre-offset; see Table 1) and was also found to increase during offset processing relative to the pre-offset base line (repeated samples t test for offset peak > pre-offset; Fig. 2B). The contrast pre-offset > offset yielded no activations. The activation patterns in all individual subjects were consistent with the group results. In 5 of the subjects we also identified activation in the inferior frontal gyrus (IFG). Overall, the source analysis data are consistent with previous reports (e.g. Fujioka et al, 2012) in suggesting that a parieto-temporal network supports the process of representing the temporal pattern in a sequence and is also activated (possibly together with IFG sources) when a violation occurs. Importantly this pattern of activation occurred even when subjects were not actively detecting the offsets.

### Experiment 2 — unattended regular but non-isochronous sequences

**Fig. 3:**
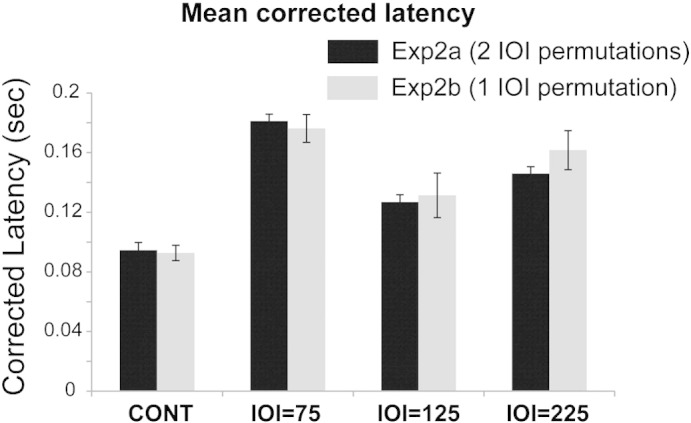
Comparison of offset latencies obtained in Experiment 2a (two possible IOI permutations) and Experiment 2b (one IOI permutation). No significant difference indicates that increased offset latency in Experiment 2 is not due to pattern variability.

**Fig. 4:**
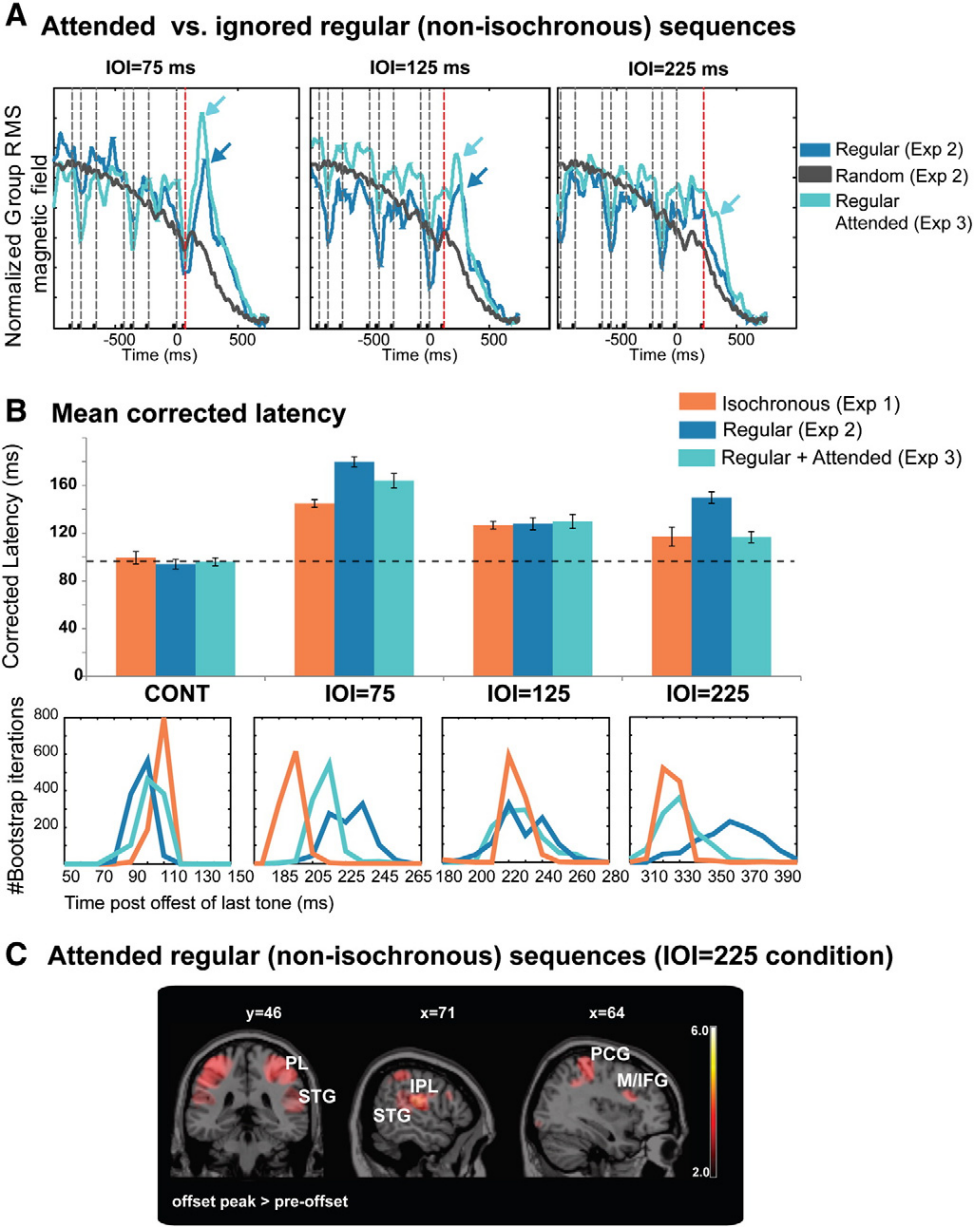
Results of Experiments 2 and 3 (regular non-isochronous sequences) A: Evoked responses (group RMS) to each of the IOI conditions (and the RAND sequence) in Experiment 2 (dark blue) and Experiment 3 (light blue). 0 ms = offset of the last tone; arrows indicate offset responses (no offset responses were visible in the grand RMS of the IOI = 225 ms condition in Experiment 2). Dashed lines indicate the presentation time of the last 8 tones in the sequence. The next expected (non-arriving) tone is shown in red. Overall the activation patterns indicate significantly delayed offset responses when the sequences are not actively attended. B: Offset response latencies across the three experiments. Top: mean latencies across subjects (corrected by subtracting relevant silent duration from raw RT). Dashed line shows the latency of the CONT conditions, for comparison. IOIs presented in the context of a regularly repeating, non-attended, pattern (Experiment 2) are associated with significantly increased offset latencies, indicating a marked reduction in coding accuracy. Once the sequences are made perceptually pertinent (though listeners were not explicitly attending to temporal structure; Experiment 3) latencies shorten considerably, approaching those measured for isochronous sequences (Experiment 1). Bottom: (raw) latency histograms computed iteratively using Bootstrap (Efron and Tibshirani, 1993). C: Localization results for the IOI = 225 ms condition in Experiment 3. Plotted are t-maps overlaid on a ch2.nii.gz atlas. Significant clusters for the offset peak > pre-offset are in superior temporal gyrus (STG), middle/inferior frontal gyrus (M/IFG) and the parietal lobe (PL) bilaterally encompassing the post-central gyrus (PCG), and the inferior parietal lobule (IPL). See also Table 2.

Experiment 2 investigated whether the auditory system is capable of learning more complex repeating patterns by using tone sequences in which different IOIs (same as those in Experiment 1) are repeated regularly (Fig. 1B). Crucially, the overall number of presentations of each IOI was the same as in Experiment 1. Two versions of the experiment were run. In the first (Experiment 2a; 15 listeners), the stimulus sequences consisted of two possible permutations of the 3 IOIs. To determine whether the delayed offset responses which were observed in this experiment (see more below), were related to the use of two different IOI orders, the experiment was repeated (control Experiment 2b; 5 listeners) using only one of the possible permutations. Overall session duration was kept constant thus Experiment 2b comprised twice as many trials of each IOI condition. These manipulations did not appear to have an effect on offset latency. A comparison of the latencies in the two versions of Experiment 2 is shown in Fig. 3. A mixed design ANOVA, with IOI condition as a within-subject factor, and version (Exp2a or Exp2b) as a between-subject factor revealed a main effect of condition (F(3,54) = 55.669; p < 0.0001) but no effect of experiment version (F(1,18) = 0.278; p = 0.604). An additional mixed design ANOVA, including the data from Experiment 1, revealed a main effect of condition (F(3,81) = 67.318; p < 0.0001) and a main effect of experiment (F(1,27) = 6.62; p < 0.0001). Post hoc, Bonferroni corrected, comparisons suggested a significant difference between Exp1 and both versions of Exp2 (p = 0.001 for both) and no difference between the two versions of Exp2 (p = 1.0). Overall, this outcome suggests that difficulty in learning non-isochronous regular sequences, manifested as delayed responses relative to those estimated for isochronous sequences, is not due to the complexity or variability of the pattern. Therefore, the latency analysis in Fig. 4 and subsequent comparisons with Experiment 3, below, pool across Experiment 2a and Experiment 2b data.

Fig. 4A (dark blue lines) shows the observed offset response pattern in Experiment 2 (for comparison with Experiment 3; see more below). The data for the CONT condition are similar to Experiment 1 and therefore not shown. The figure plots mean (grand-RMS across subjects) responses to the different IOI conditions as well as the RAND condition (in black), where the order of IOIs was random. Clear offset responses are visible in the regular IOI75 and IOI125 ms conditions (see arrows), but not in the IOI225 ms condition, where the data revealed substantial inter-listener variability (see also histogram plots in Fig. 4B) and hence no stable peak in the group-RMS. An offset peak is also not visible in the RAND condition (as expected, since these sequences lack a predictable temporal structure).

Fig. 4B displays the mean peak latencies and mean peak latency distributions across conditions and experiments (see Materials and methods). A comparison between the results of Experiment 2 (dark blue lines) and Experiment 1 (Orange lines) suggests that while there is no difference in response latencies in the CONT condition, offset peak latencies for IOI75 and IOI225 are significantly increased. Consistent with this observation, a mixed design repeated measures ANOVA on offset latencies, with experiment (Exp1 and Exp2) as between-subjects factor and condition as within-subject factor demonstrated a main effect of condition (F(3,84) = 59.333; p < 0.001), and an interaction between experiment and condition (F(3,84) = 8.969; p < 0.001). An independent samples t test confirmed significant difference between the IOI75 and IOI225 conditions across the two experiments (p < 0.001 and p = 0.001, respectively) and no difference for the other two conditions CONT and IOI125; p > 0.434).

Hence, the results suggest that, at least when the signals are not behaviourally relevant, cortical offset responses reveal sluggish learning of temporal structure in (relatively simple) non-isochronous, regular sequences. It must be stressed that offset responses are still generated by most subjects in most conditions, indicating that *some* learning of sequence structure does take place automatically but that interval coding (and hence ability to rapidly detect the offset of a sequence) is markedly less precise than that for isochronous sequences. In both the IOI75 and IOI225 conditions, offset responses are slower (relative to the isochronous conditions in Experiment 1) by about 30–40 ms and cross-subject latency distributions are wider (Fig. 4B).

Notably, performance on the IOI125 condition is relatively preserved (Fig. 4B; Experiment 1 and Experiment 2 mean latencies are not significantly different, despite some widening in the cross-subject latency distribution). This is likely a result of the fact that this interval is accentuated in our sequences and therefore more perceptually salient than the other two IOIs (more below).

### Experiment 3 — attended regular non-isochronous sequences

This experiment investigated IOI coding when the sequences are made behaviourally relevant. The same regular sequences as in Experiment 2, above, were used but in the context of a task where listeners were required to monitor the tone pips for occasional frequency deviants (Fig. 1D). Importantly, attention was not directed to sequence offset but rather equally distributed over the entire duration and the detection task was an *implicit timing* task (Coull and Nobre, 2008), not requiring overt processing of IOI duration. The mean hit rate was 88% (stde = 2.8%), with a low false positive rate (0.4%, stde = 0.16%). Group-RMS offset responses are plotted in Fig. 4A (light blue) and mean offset response latencies are in Fig. 4B (light blue). Clear mean offset responses are observed in all IOI conditions (see light blue arrows in Fig. 4A) and a comparison with the data from Experiment 2 suggests that these occur significantly earlier. A mixed design repeated measures ANOVA on offset peak latencies, with experiment (Experiment 2 vs. Experiment 3) as a between subjects factor and condition as within-subject factor demonstrated a main effect of condition (F(3,90) = 97.986; p < 0.001), and an interaction between experiment and condition (F(3,90) = 7.001; p < 0.001). An independent samples t test confirmed significant difference between the IOI75 and IOI225 conditions across the two experiments (p = 0.034 and p < 0.001, respectively) and no difference in the CONT and IOI125 conditions (p = 0.741 and p = 0.806, respectively). This pattern of results suggests, therefore, that when the sequences are made perceptually relevant, coding of temporal structure improves significantly, resulting in faster offset detection.

The procedure for source analysis is identical to that described for Experiment 1. The analysis identified 3 cortical foci which contribute to the offset peak (i.e. more active at the time of the offset peak, relative to a pre-offset interval; Fig. 4C): The Superior temporal gyrus (STG) in auditory cortex, postcentral gyrus (PCG) in the parietal lobe extending onto the inferior parietal lobule (IPL) and the precentral gyrus (M1), and the middle frontal gyrus (MFG) extending onto the inferior frontal gyrus (IFG). Activation in those areas contributed to coding the ongoing sequence (see Table 2) and was also found to increase at offset relative to the pre-offset interval. Overall the cortical network identified for the regular sequences is very similar to the one outlined for isochronous sequences in Experiment 1 (and in e.g. Fujioka et al., 2012). An important caveat is, however, that MEG tends to be relatively insensitive to deep (sub-cortical) sources, and these might be differentially activated for isochronous and non-isochronous sequences (Grahn, 2012).

### Comparison across the three experiments

A grand ANOVA across the three experiments revealed an interaction between experiment and condition (F(4.923,95.989) = 6.763; p = 0.002). A post hoc (Bonferroni corrected) test demonstrated a significant difference between Experiments 1 and 2 (p = 0.032) and between Experiments 2 and 3 (p = 0.003) with no difference between Experiments 1 and 3 (p = 1.0). Similarly, a series of univariate ANOVAs (for each condition separately) demonstrated no effect of experiment on CONT (F(2,39) = 0.377; p = 0.689), and IOI125 (F(2,39) = 0.076; p = 0.927) but a significant effect on IOI75 (F(2,39) = 13.074; p < 0.0001) and IOI225 (F(2,39) = 12.712; p < 0.0001). Post hoc tests for IOI75 show a significant difference between Exp1 and Exp2 (p = 0.02) and a significant difference between Exp1, Exp2 and Exp3 (p = 0.016 and p < 0.0001, respectively) indicating that while the mean latency in Exp3 is closer to that in Exp1 brain responses are still sluggish in that condition (attended regular sequences) relative to isochronous sequences. For IOI225, post hoc tests show a significant difference between Exp1 and Exp2 (p < 0.0001) and Exp2 and Exp3 (p < 0.0001) but no difference between Exp1 and Exp3 (p = 0.957), suggesting that attention restored the interval estimates back to those measured for isochronous sequences. This pattern is also evident in the latency distribution plots (Fig. 4B, bottom), computed using bootstrap resampling (see Materials and methods).

The attentional demands of the visual decoy task are low. The images are presented at a measured rate and their comparison is straightforward — leaving ample perceptual/computational resources to devote to the auditory stimuli. Despite this, the pattern of results across Experiments 2 and 3 suggests that accurate temporal pattern learning takes place only when the acoustic sequences are *actively* attended.

That CONT offset latencies are constant across experiments is perhaps not surprising — simple (first order) offset responses likely rely on automatic processes (Phillips et al., 2002; Scholl et al., 2010) and hence not affected by attention. However, the finding that the IOI125 condition was not affected by these manipulations is not trivial. A likely explanation is that, due to the beat structure of the regular sequences, this interval was subjectively accentuated and therefore ‘popped out’ even in Experiment 2, when the sequences were not actively attended, resulting in accurate duration coding (indeed such an account is consistent with the 3 stage clock model; Brochard et al., 2003; see also Povel and Essens, 1985; Povel and Okkerman, 1981).

## Discussion

### Sensitivity to time

Accumulating evidence demonstrates that listeners, including new-borns (Winkler et al., 2009), are sensitive to the timing of sound sequences and form expectancies about future temporal events. When exposed to on-going regular patterns, brain responses reflect temporal orienting in preparation to process expected events (Lange, 2013; but see Schwartze et al., 2011). Behaviourally, the consequence of these processes is that expected events (within a regular temporal context) are responded to more rapidly than those occurring in a temporally irregular pattern (Barnes and Jones, 2000; Ellis and Jones, 2010) and are associated with increased sensitivity (Geiser et al., 2012; Jones, 1976; Tavano et al, 2014). Imaging work (though using relatively simple sound patterns) suggests that this sensitivity does not necessarily require explicit attention to sequence timing (Hughes et al., 2001; Karamürsel and Bullock, 2000; Yamashiro et al., 2009). Indeed, the preceding temporal context often affects performance even when it is in the listeners' best interest to ignore it.

In theory, and especially within the framework of predictive-coding, recently attracting considerable attention (Wacongne et al., 2011; Nelken, 2012; Clark, 2013; Friston, 2005), any regularly repeating temporal structure should be learnable as long as the system has the capacity, and sufficient opportunity, to accumulate the relevant statistics. Probing the limits of this learning, viz. understanding which temporal patterns are acquired and under what conditions, should thus provide a key handle onto the neural computations underlying sensitivity to time. However, work thus far has used only very simple (mostly isochronous) and slow (< 4 Hz) temporal patterns. The purpose of this series of experiments was to measure sensitivity to temporal patterning in non-isochronous sequences and at fast rates, pertinent to natural sources (Rosen, 1992; Shamma and Micheyl, 2010; Andreou et al., 2011).

The paradigm is based on probing brain responses to offsets of tone-pip sequences. Using simple (isochronous) regular sequences and a more complex variant where three different inter-onset-intervals alternate in regular succession, we demonstrate that offset responses — deflections occurring after the non-arrival of an expected tone — are present in the evoked response (see also, Yamashiro et al., 2009). These deflections, or ‘second order transients’ are specific to regular patterning (not observed in a random control sequence) suggesting that the auditory system is sensitive to the temporal structure of on-going sound input and registers when it is violated. It is hypothesized that the neural computations supporting this sensitivity involve learning the order of IOIs in the sequence, anticipating upcoming IOIs, and signalling a violation when an expected tone fails to arrive.

### Offset responses as indicators of temporal pattern learning

The pattern of offset latencies over the three experiments reported here demonstrates that: (a) Overall, the brain consistently underperforms for the shortest (IOI75 ms) interval (see also Zanto et al., 2006), as reflected in relatively (compared to the other IOI conditions) long corrected latencies and weaker facilitation by attention. This is despite that many natural sounds, including speech, contain, what is thought to be, perceptually relevant temporal information in this range. (b) IOIs presented in the context of a regularly repeating pattern are associated with significantly increased offset latencies, relative to IOIs within an isochronous sequence, indicating a marked reduction in coding accuracy. (c) Once the sequences are made perceptually pertinent (and even when temporal structure is not explicitly attended) latencies shorten significantly, approaching those measured for isochronous sequences.

Source analysis revealed a network of temporal (focus around STG), frontal (MFG, IFG) and parietal (encompassing postcentral gyrus and the inferior parietal lobule) sources in coding the temporal properties of ongoing sound sequences and their violation. This is consistent with findings in the neuroimaging (Grahn, 2012; Chen et al., 2008a; Martin et al., 2008; Coull and Nobre, 2008), intra-cranial recordings (Hughes et al., 2001) and electrophysiology (Leon and Shadlen, 2003; Rao et al., 2001; Janssen and Shadlen, 2005) literature, indicating an association of parietal cortex with temporal processing. MFG/IFG activations are often observed in the context of deviance detection (Tse et al., 2006; Doeller et al., 2003; Schönwiesner et al., 2007) and expectancy (e.g. Osnes et al., 2012; Doricchi et al., 2010; Seger et al., 2013; Grahn, 2012) as well as working memory and rule generation (Chen et al., 2008b) — all processes that are consistent with computations required for offset detection. Importantly, our results suggest that isochronous and non-isochronous regular sequences involve processing in the same cortical foci (with a caution that sub-cortical structures, which might be differentially engaged, are difficult to resolve with MEG; Grahn, 2012; Grahn and Rowe, 2009).

In contrast to many reports of motor cortex activations during passive listening to temporal patterns (Grahn, 2012; Fujioka et al., 2012; Zatorre et al., 2007) only a small extent of such activation was recorded here. This might be because motor cortex involvement, which is thought to reflect sensory-motor synchronization (e.g. for motor planning), only occurs for slower rates (IOI > 200 ms), above the biomechanical limit. The rates here were mostly outside of this range.

### Representation of time in the auditory brain

Various mechanisms have been proposed to account for temporal structure learning (Coull, 2009; Grahn, 2012; Merchant et al., 2013). Currently receiving significant attention are a family of entrainment models (Barnes and Jones, 2000; Arnal and Giraud, 2012; Schwartze and Kotz, 2013), according to which the brain ‘locks’ to the temporal structure of the input and the resulting neural oscillations (periodic increases in excitability) underlie the effects of time-dependant performance and prediction. Recent reports are largely consistent with this premise. Fujioka et al. (2012) demonstrated periodic modulation in the beta-band that anticipates the occurrence of repeatedly presented tone-pips (see also Snyder and Large, 2005). Lakatos et al. (2013), using neuronal ensemble recordings in A1 of behaving monkeys, recorded sub-threshold oscillatory activity reflecting entrainment to an isochronous sequence. The dynamics of that response were very similar to those observed in humans by Fujioka et al. (2012).

Interestingly, Lakatos et al. (2013), revealed that neural oscillations entrained to an isochronous tone-pip sequence continue to exhibit structured rhythmic excitability fluctuations for at least 4 s after the sequence has ended, with no evidence of offset responses. This suggests that the offset response (= change detection) system is, to some extent, decoupled from the entrainment mechanism, consistent with our localization results which identified sources in STG (non-primary auditory cortex) but not in A1.

It is presently unclear how the mechanisms identified by e.g. Lakatos et al. (2013), might extend to non-isochronous regular sequences as used here. One possibility is that acquisition is accomplished by a number of independent oscillators, all with the same period but a different phase such that each is entrained to successive tones in the pattern, resulting in *implicit* coding of temporal order. MEG is unlikely to be sufficiently sensitive for revealing such oscillator populations but they should be discernible with electrophysiological means. Alternatively, the observed learning could be accomplished within ‘classically postulated’ clock-based interval timing mechanisms (Gibbon et al., 1984; Keele et al., 1989; Rao et al., 2001; Teki et al., 2011) coding interval duration (and their order) explicitly.

Isochronous sequences, or those confirming to an exact metrical hierarchy, possess a special perceptual status (Grube and Griffiths, 2009), possibly underpinned by an automatic striato–thalamo-cortical network (Teki et al., 2011; Grahn and Rowe, 2009). It has been hypothesized that non-metrical regular sequences are not represented precisely but ‘regularized’ toward the nearest metrical pattern (Motz et al., 2013). However, the present data are not consistent with such an account because the same sequences, when made perceptually pertinent, are associated with nearly accurate coding. Instead, our data suggest that the limiting factor might not be a computational one but rather that, save for isochronous patterns, listeners only precisely acquire temporal structure when it is *immediately* perceptually relevant. Thus, perhaps surprisingly, timing information despite its key importance for predicting event occurrence and facilitating efficient interaction with one's surroundings, does not appear to be extracted robustly when outside of the focus of attention.

## Funding

This project was funded by a Deafness Research UK fellowship and a Wellcome Trust project grant (093292/Z/10/Z) to Maria Chait.

## Figures and Tables

**Table 1:** Source localization results for Experiment 1.

Brain area	Hemisphere	*x*	*y*	*z*	*t*-Value	p
Experiment 1, pre-offset						
Parietal lobe, postcentral gyrus	Left	− 40	− 38	52	4.55	0.001
Parietal lobe, postcentral gyrus	Left	− 38	− 38	60	4.55	0.001
Parietal lobe, postcentral gyrus	Left	− 60	− 18	24	4	0.002
Parietal lobe, supramarginal gyrus	Left	− 58	− 50	26	4.73	0.001
Temporal lobe, superior temporal gyrus	Left	− 60	− 32	14	4.13	0.001
Occipital lobe, cuneus	Left	− 2	− 96	4	3.91	0.002
Occipital lobe, cuneus	Left	− 6	− 80	30	3.07	0.007
Parietal lobe, postcentral gyrus	Right	38	− 36	60	4.92	0
Parietal lobe, postcentral gyrus	Right	60	− 22	26	4.01	0.002
Temporal lobe, superior temporal gyrus	Right	54	− 36	14	4.85	0
Temporal lobe, superior temporal gyrus	Right	62	− 38	16	4.7	0.001
Occipital lobe, cuneus	Right	8	− 84	32	3.08	0.007

*Experiment 1, post-offset*						
Parietal lobe, postcentral gyrus	Left	− 38	− 38	60	2.86	0.009
temporal lobe, superior temporal gyrus	Left	− 56	− 40	14	3.49	0.003
Parietal lobe, postcentral gyrus	Right	38	− 36	60	2.86	0.009
Temporal lobe, superior temporal gyrus	Right	54	− 32	6	3.5	0.003

*Experiment 1, post-offset* > *pre-offset*						
Temporal lobe, superior temporal gyrus	Left	− 56	− 40	14	3.11	0.006
Parietal lobe, postcentral gyrus	Right	34	− 38	60	2.26	0.025
Temporal lobe, superior temporal gyrus	Right	56	− 34	6	2.41	0.02

**Table 2:** Source localization results for Experiment 3.

Brain area	Hemisphere	*x*	*y*	*z*	*t*-Value	p
*Experiment 3, pre-offset*						
Parietal lobe, postcentral gyrus	Left	− 52	− 26	14	6.74	0
Parietal lobe, postcentral gyrus	Left	− 64	− 14	18	4.27	0.001
Parietal lobe, precuneus	Left	− 18	− 78	38	2.8	0.009
Parietal lobe, sub-gyral	Left	− 38	− 34	48	6.36	0
Frontal lobe, precentral gyrus	Left	− 28	− 32	56	6.43	0
Frontal lobe, superior frontal gyrus	Left	− 8	− 10	68	4.43	0.001
Occipital lobe, lingual gyrus	Left	− 6	− 88	− 10	4.59	0
Occipital lobe, lingual gyrus	Left	− 20	− 78	− 10	4.28	0.001
Occipital lobe, cuneus	Left	− 8	− 80	30	2.84	0.008
Parietal lobe, sub-gyral	Right	38	− 36	48	5.82	0
Temporal lobe, superior temporal gyrus	Right	62	− 38	20	4.3	0.001
Frontal lobe, precentral gyrus	Right	62	− 8	12	5.06	0
Frontal lobe, medial frontal gyrus	Right	8	− 14	68	4.43	0.001
Frontal lobe, middle frontal gyrus	Right	30	28	38	2.97	0.006
Frontal lobe, inferior frontal gyrus	Right	50	26	0	2.33	0.02
Occipital lobe, lingual gyrus	Right	16	− 80	− 12	4.29	0.001
Occipital lobe, cuneus	Right	10	− 78	30	2.97	0.006

*Experiment 3, post-offset*						
Parietal lobe, postcentral gyrus	Left	− 36	− 32	56	4.87	0
Parietal lobe, postcentral gyrus	Left	− 50	− 24	16	4.99	0
Parietal lobe, precuneus	Left	− 18	− 80	40	2.79	0.009
Parietal lobe, sub-gyral	Left	− 40	− 34	46	3.7	0.002
Temporal lobe, middle temporal gyrus	Left	− 56	− 14	− 14	4.54	0
Frontal lobe, precentral gyrus	Left	− 26	− 32	58	5.21	0
Frontal lobe, middle frontal gyrus	Left	− 46	20	30	2.68	0.011
Frontal lobe, sub-gyral	Left	− 38	18	26	2.68	0.011
Frontal lobe, superior frontal gyrus	Left	− 8	− 10	68	2.48	0.015
Occipital lobe, lingual gyrus	Left	− 8	− 90	− 12	3.45	0.003
Occipital lobe, lingual gyrus	Left	− 18	− 76	− 12	3.04	0.006
Parietal lobe, postcentral gyrus	Right	52	− 18	16	4.45	0
Parietal lobe, sub-gyral	Right	26	− 40	52	3.76	0.002
Parietal lobe, inferior parietal lobule	Right	48	− 48	46	3.07	0.005
Parietal lobe, supramarginal gyrus	Right	58	− 40	30	3.14	0.005
Parietal lobe, precuneus	Right	20	− 82	40	2.83	0.008
Frontal lobe, precentral gyrus	Right	60	− 6	14	4.52	0
Frontal lobe, middle frontal gyrus	Right	46	16	28	2.75	0.009
Frontal lobe, medial frontal gyrus	Right	8	− 14	68	2.53	0.014
Occipital lobe, lingual gyrus	Right	14	− 80	− 12	2.96	0.006

*Experiment 3, post-offset* > *pre-offset*						
Parietal lobe, postcentral gyrus	Left	− 22	− 30	58	4.59	0
Parietal lobe, postcentral gyrus	Left	− 32	− 34	54	4.12	0.001
Parietal lobe, postcentral gyrus	Left	− 54	− 24	16	4.16	0.001
Parietal lobe, postcentral gyrus	Left	− 60	− 18	20	3.8	0.001
Parietal lobe, inferior parietal lobule	Left	− 46	− 42	48	3.16	0.005
Frontal lobe, middle frontal gyrus	Left	− 46	20	30	2.7	0.01
Frontal lobe, sub-gyral	Left	− 38	18	26	2.71	0.01
Parietal lobe, precuneus	Right	20	− 82	40	2.41	0.017
Parietal lobe, postcentral gyrus	Right	60	− 20	20	3.82	0.001
Parietal lobe, postcentral gyrus	Right	22	− 30	58	2.85	0.008
Parietal lobe, sub-gyral	Right	32	− 32	48	3.13	0.005
Parietal lobe, inferior parietal lobule	Right	48	− 48	46	2.93	0.007
Temporal lobe, superior temporal gyrus	Right	58	− 34	8	2.44	0.016
Frontal lobe, precentral gyrus	Right	60	− 4	24	2.81	0.008
Frontal lobe, middle frontal gyrus	Right	46	16	28	2.77	0.009
